# CMR-Verified Myocardial Fibrosis Is Associated With Subclinical Diastolic Dysfunction in Primary Aldosteronism Patients

**DOI:** 10.3389/fendo.2021.672557

**Published:** 2021-05-14

**Authors:** Fangli Zhou, Tao Wu, Wei Wang, Wei Cheng, Shuang Wan, Haoming Tian, Tao Chen, Jiayu Sun, Yan Ren

**Affiliations:** ^1^ Adrenal Center, Department of Endocrinology and Metabolism, West China Hospital, Sichuan University, Chengdu, China; ^2^ Departments of Radiology, West China Hospital, Sichuan University, Chengdu, China

**Keywords:** primary aldosteronism, essential hypertension, left ventricular function, the global circumferential PDSR, myocardial fibrosis, CMR

## Abstract

**Objectives:**

The main cardiac features of primary aldosteronism (PA) are impaired left ventricular (LV) diastolic function, and some articles also reported more cardiac fibrosis in PA patients. However, the correlation between LV dysfunction and diffuse myocardial fibrosis in PA remains unknown.

**Methods:**

We enrolled 84 PA patients and 28 essential hypertension (EH) patients in West China Hospital. Cardiac magnetic resonance imaging (CMR) contrast enhancement was arranged for all subjects. Postcontrast T1 time and left ventricular myocardial strains and strain rates were measured.

**Results:**

76 PA patients and 27 essential hypertension (EH) patients were included in the final analysis. Blood pressure, LV mass indexes, and LV ejection fractions were comparable in both groups, while the global circumferential peak diastolic strain rate (PDSR) was lower (0.9 ± 0.3 vs. 1.1 ± 0.4, p <0.01) and the postcontrast T1 time was shorter (520 ± 38 vs. 538 ± 27, p = 0.01) in PA patients than those in EH patients. Postcontrast T1 time (p = 0.01) was independently related to global circumferential PDSR after adjusting for age and duration of hypertension in PA patients. Furthermore, plasma aldosterone concentration was negatively associated with postcontrast T1 time (R = −0.253, p = 0.028) in PA patients.

**Conclusions:**

The global circumferential PDSR derived by CMR is decreased, and the diffuse myocardial fibrosis is increased in PA patients compared to those in blood pressure matched EH patients. The severity of cardiac diastolic dysfunction independently relates to the degree of diffuse myocardial fibrosis in PA patients, and the diffuse myocardial fibrosis may be caused by high PAC level.

**Clinical Trial Registration:**

http://www.chictr.org.cn/listbycreater.asp, identifier ChiCTR2000031792.

## Introduction

Primary aldosteronism (PA), characterized by the overproduction of aldosterone that seems autonomous from renin (1), is the most common endocrine cause of hypertension and contributes more than 10% to the etiology of hypertension (2) and 29.1% to the etiology of resistant hypertension (3). In addition to high blood pressure, increased aldosterone, which has proinflammatory (4) and profibrotic effects (5) on myocardial tissues, is another risk factor for cardiovascular diseases. Previous clinical studies have shown that PA patients are prone to cardiovascular complications (6–9). PA patients revealed a significantly higher prevalence of coronary artery disease (adjusted OR, 1.9), nonfatal myocardial infarction (adjusted OR, 2.6), heart failure (adjusted OR, 2.9), and atrial fibrillation (adjusted OR, 5.0) than essential hypertension (EH) patients (6). Moreover, Reincke et al. (8) reported that cardiovascular mortality was the leading cause of death in PA (50%) and occurred less frequently in EH controls (34%).

It is well known that PA patients’ major cardiac damage is impaired left ventricular function (10, 11), leading to poor prognosis in PA patients. The pathophysiological mechanism of left ventricular dysfunction in PA patients remains unclear. Studies have found that cardiac dysfunction results from fibrosis of the myocardium in many diseases, such as heart failure (12), hypertrophic cardiomyopathy (13), and diabetic patients (14). As to if myocardial fibrosis increases in PA patients, the answer is still controversial (15–19). Cardiac biopsy samples from four male PA patients exhibited 1.5-fold more fibrosis than those from EH patients (14% vs. 6%) (18). A late gadolinium enhancement study using cardiac magnetic imaging (CMR) proved that patients with PA exhibit more frequent diffuse myocardial fibrosis than healthy volunteers (16) and EH patients (17). Moreover, adrenalectomy was proven to reverse myocardial fibrosis in PA patients (19). However, Gretaas et al. (15) reported that increased myocardial fibrosis was not found and may not represent a common clinical problem in PA.

CMR offers a non-invasive, highly accurate assessment of cardiac function and geometry, and contrast-enhanced T1 mapping can provide evidence of diffuse myocardial fibrosis (14, 20). Post-contrast myocardial T1 time is inversely correlated with histologically defined interstitial fibrosis, so shorter postcontrast T1 time represents more interstitial fibrosis (21, 22). However, the correlation of left ventricle (LV) dysfunction and myocardial fibrosis in PA patients remains unknown. Thus, the aims of the prospective observational study were as follows: (1) to compare global postcontrast T1 time between PA patients and age-, sex-, body mass index-, blood pressure-, and hypertension duration-controlled EH subjects; and (2) if the PA patients own shorter postcontrast T1 time, we will try to explore the relationship between cardiac left ventricular function and myocardial fibrosis in PA patients.

## Materials and Methods

### Study Population

From April 2018 to May 2019, 84 PA patients were recruited from the inpatient department of Endocrinology and Metabolism of West China Hospital. 28 EH patients were recruited from the medical examination center of West China Hospital. All the subjects were of Han ethnicity and between 22 and 78 years old. All the patients completed the initial screening test of the plasma aldosterone/renin ratio (ARR) for PA, plasma renin activity (PRA) <0.2 ng/ml/h was set as 0.2 for the calculation of the ARR to avoid inflation due to a very low denominator. According to the guidelines, before the initial screening, all the subjects had been on stable antihypertensive treatment with an α1-adrenergic receptor antagonist alone or verapamil sustained-release agent or hydralazine, and with normal serum potassium levels. The duration of stable antihypertensive treatment depended on the antihypertensive drugs the patients were taking. MR antagonists (e.g., spironolactone, eplerenone), potassium-sparing diuretics (e.g., amiloride, triamterene), and potassium-wasting diuretics (e.g., hydrochlorothiazide, furosemide) should be withdrawn for at least four weeks before ARR testing. Angiotensin-converting enzyme inhibitors, angiotensin receptor blockers, renin inhibitors, dihydropyridine calcium channel antagonists, β-Adrenergic blockers, and central α-2 agonists (e.g., clonidine, α-methyldopa) should be withdrawn for at least two weeks before ARR testing. The PA patients were further diagnosed by confirmatory tests of saline infusion and/or captopril challenge according to current guideline (23). The exclusion criteria included the following: ① subjects with a history of congestive heart failure, chronic steroid therapy or chronic kidney disease (estimated glomerular filtration rate <60 ml/min); ② subjects with clinical indications of other secondary causes of hypertension except for PA, such as renal artery stenosis, pheochromocytoma, Cushing’s syndrome, and hyperthyroidism.

This study was revised and confirmed by the Ethics Committee of West China Hospital. Before the study, written informed consent was obtained from each individual. Our study was registered in the Chinese clinical trial registry (ChiCTR2000031792).

### Demographic Characteristics and Laboratory Determinations

Two trained staff members recorded the clinical characteristics of subjects with a standard questionnaire. Physical examination was performed on all the subjects, including body height, weight, waist circumference, and blood pressure. Body mass index (BMI) was computed as body weight in kilograms (kg) divided by height in meters squared (m2). The body surface areas were calculated as 0.0057 × height (cm) + 0.0121 × weight (kg) + 0.0882 for males and 0.0073 × height (cm) + 0.0127 × weight (kg) − 0.2106 for females. All patients underwent 24-hour ambulatory blood pressure monitoring three days before the CMR scan.

After overnight fasting (≥8 h), venous blood samples were collected to measure plasma aldosterone concentration (PAC), PRA, and other biochemical parameters. Radioimmunoassay was used to measure PAC and PRA (Beijing North Institute of Biotechnology Co., St. Panjia Miao, Beijing). Serum potassium, serum sodium, total cholesterol, triglycerides, low-density lipoprotein cholesterol, high-density lipoprotein cholesterol, pro-brain natriuretic peptide (pro-BNP), creatine kinase MB, troponin T, and myoglobin concentrations were measured for all patients using automated, standardized equipment by the Clinical Laboratory at West China Hospital.

### Cardiovascular Magnetic Resonance Protocol

CMR was performed with a 3.0 T MRI imager (Trio Tim; Siemens Medical Solution, Erlangen, Germany) using an eight-channel phased-array surface coil and prospective electrocardiographic triggering. Patients assumed a supine position, and ECG gating and respiratory gating were used throughout the scan. The cine images of the short-axis covering the LV and the long-axis (two-, three- and four-chamber views) were obtained with a segmented balanced steady-state-free-precession sequence. The scanning parameters were TR/TE 3.4/1.3 ms, flip angle 50°, a field of view 320–340 mm, matrix size 256 × 144, and section thickness, 8 mm with no gap.

**Figure 1:**
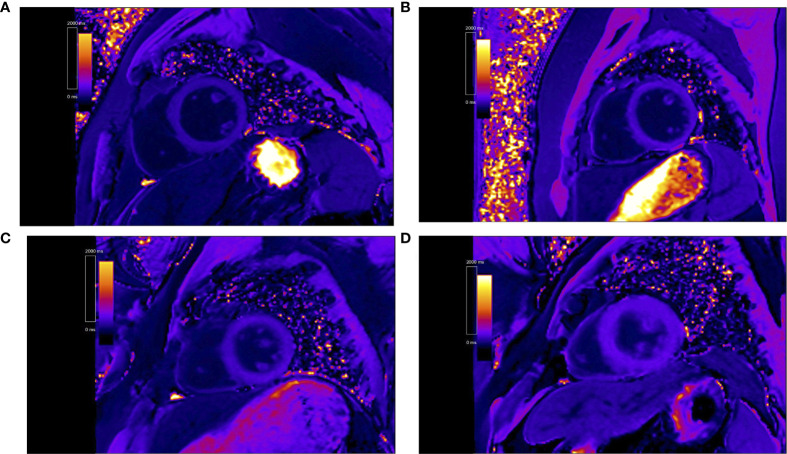
Representative contrast-enhanced T1 maps of primary aldosteronism patients [**(A)** patient 1, post T1 of 451.1 ms. **(B)** patient 2, post T1 of 426.7 ms] and essential hypertension patients [**(C)** patient 3, post T1 of 568.4 ms. **(D)** patient 4, post T1 of 589.5 ms].

Postcontrast T1 maps were acquired using modified look-locker inversion recovery sequence (total acquisition is 17 heartbeats, TR/TE 2.9/1.12 ms, 8 mm thickness, in-plane spatial resolution 2.4 mm × 1.8 mm, matrix size 192 × 144, flip angle 35°, bandwidth 930 Hz/pixel, TI of first experiment 100 ms, TI increment 80 ms, parallel imaging 2), which was recommended by the Society for Cardiovascular Magnetic Resonance (24). Postcontrast T1 mapping was performed 15 min after the intravenous bolus injection administration of gadobenate dimeglumine (MultiHance; 0.5 mmol/ml; Bracco, Milan, Italy) at a dose of 0.15 mmol/kg body weight. About the gadolinium-based contrast doses, 0.1–0.2 mmol/Kg is recommended for T1 mapping in the European Association for Cardiovascular Imaging recommendation (24). Our research center used 0.15 mmol/kg to detect T1 mapping for many years. Some researches have been reported (25–27). **Figure 1** shows the contrast-enhanced T1 maps of two PA patients and two EH patients.

### CMR Data Analysis

**Figure 2:**
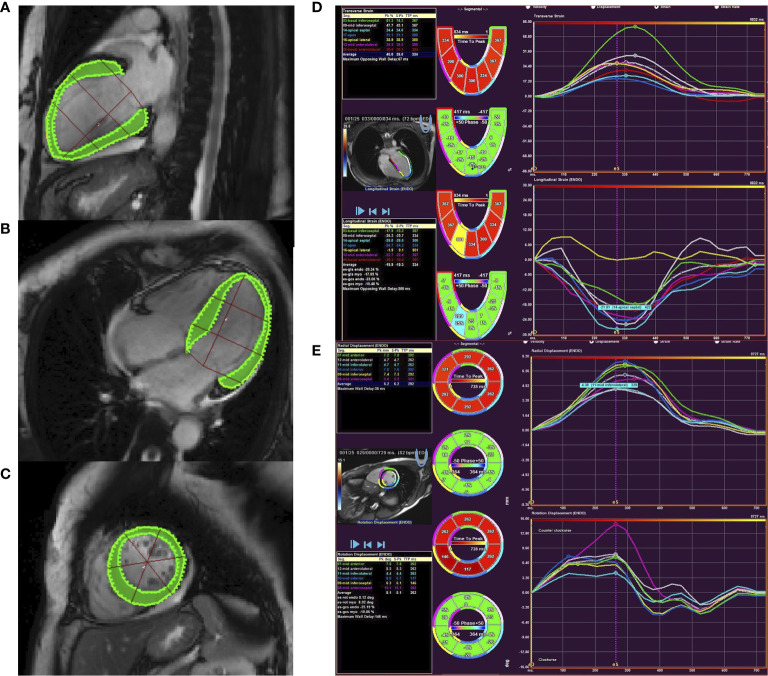
Cardiac magnetic resonance feature tracking of the left ventricle in PA patient. Panels **(A–C)** showed countering for LV longitudinal **(A)**, circumferential **(B)**, and radial **(C)** strain and strain rate. Panel **(D)** showed tracking in the short-axis image with circumferential and radial strain curves. Panel **(E)** showed tracking from the four-chamber image to derive longitudinal strain.

All imaging data of PA patients and EH subjects were uploaded to Argus software (Siemens Healthcare, Erlangen, Germany). Two experienced radiologists blinded to clinical data defined the end-diastole, end-systole, and delineated LV endocardial and epicardial borders (**Figure 2**). LV end-diastolic volume, LV end-systolic volume, LV mass, and LV ejection fraction were then calculated, and volumes and mass were indexed to body surface area. The software also automatically calculated the global myocardial strain parameters, including radial, circumferential, and longitudinal peak strain (PS), peak systolic strain rate (PSSR), and peak diastolic strain rate (PDSR), and the intra- and inter-observer variability were calculated. To evaluate diffuse myocardial fibrosis, post-processing software (Qmass 7.6; Medis, The Netherlands) was used to measure the value of myocardial native T1 time and postcontrast T1 time at the mid-layer myocardium of left ventricular basal, middle and apical segments. To attain blood T1, the regions of interest in the blood pool of the LV cavity in pre- and post-contrast T1-mapping images were drawn. ECV was calculated as: ECV= (1 − hematocrit) × (ΔR1myocardium − ΔR1blood), in which R1 = 1/T1.

### Statistical Analysis

Normally distributed data were expressed as the mean (± SD) for continuous variables, and categorical variables were expressed as percentages. Skewed variables were logarithmically transformed before analysis and expressed as medians (interquartile ranges). The quantitative variables were compared using a t-test, while the qualitative variables were compared using the χ2 test. Univariable analysis was performed to discover the correlation of the global circumferential PDSR with postcontrast T1 time and other risk factors. The association of the global circumferential PDSR with postcontrast T1 time in PA patients was analyzed with the stepwise multivariate analysis. Variables included in the regression model were those parameters p ≤0.1 in univariable analysis. P ≤0.05 was considered statistically significant. Pearson’s analysis was used to test the relationship between PAC and postcontrast T1 time in PA patients. Analyses were performed with SPSS 17.0 (Chicago, IL) for Windows.

## Results

### Characteristics and Metabolic Parameters of PA and EH Patients

All patients successfully underwent CMR except for nine patients (eight PA patients and one EH patient) who had severe arrhythmia, causing ECG synchronization failure during the study session. Therefore, 76 PA patients and 27 EH patients were included in the analysis. **Table 1** shows the demographic characteristics and laboratory data of the subjects. Age, duration of hypertension, waist circumference, BMI, systolic blood pressure, and diastolic blood pressure did not significantly differ between the EH and PA groups. PA patients had higher PAC levels, ARR, high-density lipoprotein cholesterol, serum sodium, and pro-BNP, and lower levels of PRA and serum potassium than those in EH patients.

**Table 1:** Demographic characteristics and laboratory data of patients.

Variable	EH (n = 27)	PA (n = 76)	*P* value
**Age, year**	46 ± 15	48 ± 11	0.62
**Women, number (% in total number)**	15 (54)	54 (72)	0.16
**Duration of hypertension, month**	72 (96)	36 (114)	0.42
**Waist, cm**	89 ± 7	88 ± 10	0.80
**BMI, kg/m^2^**	25 ± 3	25 ± 4	0.50
**SBP, mmHg**	146 ± 11	145 ± 17	0.86
**DBP, mmHg**	94 ± 12	93 ± 11	0.69
**TG, mmol/L**	1.5 (0.9)	1.3 (0.9)	0.09
**TC, mmol/L**	4.3 ± 1.0	4.5 ± 0.9	0.42
**LDL-C, mmol/L**	2.5 ± 0.8	2.7 ± 0.7	0.29
**HDL-C, mmol/L**	1.2 ± 0.4	1.4 ± 0.4	0.04
**PAC, ng/dl**	23 ± 9	32 ± 12	<0.01
**PRA, ng/ml/h**	3.0 (2.7)	0.2 (0.5)	<0.01
**ARR, IU/L**	9 (6)	102 (104)	<0.01
**K, mmol/L**	4.0 ± 0.4	3.5 ± 0.6	<0.01
**Na, mmol/L**	141.7 ± 1.6	142.8 ± 2.2	0.02
**Pro-BNP, pg/ml**	45 (38)	76 (93)	<0.01
**CKMB, ng/ml**	1.0 (0.5)	1.1 (0.7)	0.32
**TPN-T, ng/L**	6 (4)	7 (6)	0.64
**Myo, ng/ml**	28 (15)	29 (11)	0.54
**Number of antihypertensive medications**	2.2 ± 0.9	2.0 ± 1.1	0.35
**Number (% in total number)**			
**CCB**	24 (89)	59 (78)	0.20
** ACEI**	2 (7)	4 (5)	0.68
** ARB**	10 (37)	34 (45)	0.49
** Diuretics**	2 (7)	9 (12)	0.52
** α-blocker**	7 (26)	15 (20)	0.50
** β-blpcker**	11 (41)	19 (25)	0.12
** MR antagonists**	2 (7)	8 (11)	0.64

BMI, body mass index; SBP, systolic blood pressure; DBP, diastolic blood pressure; TC, total cholesterol; TG, triglycerides; HDL-C, high-density lipoprotein cholesterol; LDL-C, low-density lipoprotein cholesterol; PAC, plasma aldosterone concentration; PRA, plasma renin activity; ARR, aldosterone-to-renin ratio; Pro-BNP, pro-brain natriuretic peptide; CKMB, creatine kinase MB; TPN-T, troponin T; Myo, myoglobin; PTH, parathyroid hormone; CCB, calcium channel blockers; ACEI, angiotensin-converting enzyme inhibitors; ARB, angiotensin II receptor blockers.

### CMR Data in PA and EH Patients

CMR results for LV mass, volumes, function, and contrast-enhanced T1 mapping are summarized in **Table 2**. No significant differences were found in LV mass index, LV end-diastolic volume index, LV end-systolic volume index, and LV ejection fraction between PA patients and EH patients. As to T1 mapping, PA patients possessed significantly shorter post-T1 time (520 ± 38 vs. 538 ± 27, p = 0.01), higher native T1 (1,228 ± 40 vs. 1,196 ± 44, p <0.01) and ECV (27 ± 4 vs. 25 ± 3, p = 0.02) than EH patients. Data on LV function are also presented in **Table 2**. The global circumferential PDSR (0.9 ± 0.3 vs. 1.1 ± 0.4, p <0.01) was decreased in PA patients than EH patients. Other LV function parameters, including the global radial, circumferential, and longitudinal PS, PSSR, and radial, longitudinal PDSR, did not show significant differences between the two groups.

**Table 2:** Comparison of CMR results between EH and PA patients.

Variable	EH (n = 27)	PA (n = 76)	*P* value
**LV massi, gm/m^2^**	58 ± 11	59 ± 16	0.87
**LVEDVi, ml/m^2^**	79 ± 14	81 ± 15	0.44
**LVESVi, ml/m^2^**	32 ± 9	34 ± 11	0.49
**LVEF, %**	62 ± 11	59 ± 8	0.26
**Native T1 time, ms**	1,196 ± 44	1,228 ± 40	<0.01
**Postcontrast T1 time, ms**	538 ± 27	520 ± 38	0.01
**ECV, %**	25 ± 3	27 ± 4	0.02
**PS, %**			
** Radial**	44 ± 7	43 ± 9	0.64
** Circumferential**	−15 ± 2	−15 ± 3	0.84
** Longitudinal**	−14 ± 2	−14 ± 3	0.80
**PSSR, 1/s**			
** Radial**	3.7 ± 0.8	3.4 ± 0.9	0.26
** Circumferential**	−1.5 ± 0.3	−1.5 ± 0.4	0.47
** Longitudinal**	−1.3 ± 0.3	−1.2 ± 0.3	0.19
**PDSR, 1/s**			
** Radial**	−2.7 ± 1.3	−2.2 ± 1.0	0.07
** Circumferential**	1.1 ± 0.4	0.9 ± 0.3	<0.01
** Longitudinal**	1.0 ± 0.3	1.0 ± 0.3	0.97

LVEDVi, Left ventricular end-diastolic volume index; LVESVi, left ventricular end-systolic volume index; LV massi, left ventricular mass indexed to body surface area; LVEF, left ventricular ejection fraction; PS, peak strain; PSSR, peak systolic strain rate; PDSR, peak diastolic strain rate.

### Factors Affecting Global Circumferential PDSR in PA Patients

To further identify the parameters affecting global circumferential PDSR in PA patients, we used univariate analysis to demonstrate the relationship between global circumferential PDSR and potentially related factors. Global circumferential PDSR statistically related to age (R = 0.39, p <0.01), duration of hypertension (R = −0.21, p = 0.08) and postcontrast T1 time (R = 0.30, p = 0.01).

### Independent Determinants of LV Global PDSR in PA Patients

Parameters that are p ≤0.1 (**Table 3**) in univariable analysis were included in the multiple linear regression model. Specifically, age, hypertension duration, and postcontrast T1 time were brought into the multiple linear regression of global circumferential PDSR. **Table 4** showed the multiple linear regression results, which demonstrated that postcontrast T1 time was independently associated with the global circumferential PDSR (β = 0.257, p = 0.012, model R = 0.593) after adjusting for age and duration of hypertension.

**Table 3:** Univariable linear regression analysis for global circumferential PDSR in PA patients.

Variable	Correlation Coefficient	*P* value
**Age**	0.39	<0.01
**Gender**	−0.14	0.22
**Ln duration of hypertension**	−0.21	0.08
**BMI**	0.11	0.37
**SBP**	0.05	0.68
**DBP**	−0.08	0.48
**HDL-C**	0.15	0.19
**K**	−0.17	0.15
**Na**	−0.05	0.68
**PAC**	0.07	0.58
**Ln PRA**	0.15	0.21
**Ln ARR**	−0.14	0.25
**Ln Pro-BNP**	−0.02	0.89
**Native T1 time**	−0.08	0.51
**Postcontrast T1 time**	0.30	0.01
**ECV**	0.04	0.71

Abbreviations as in **Tables 1** and **2**.

**Table 4:** Independent determinants of LV global PDSR in PA patients.

Variable	Unstardardized β	95% CI	*P* value
**Age**	0.994	0.619-1.369	<0.001
**Ln duration of hypertension**	−3.829	−6.371–1.287	0.004
**Postcontrast T1 time**	0.138	0.031–0.245	0.012

Abbreviations as in **Table 2**.

### Postcontrast Myocardial T1 Time Negatively Related to PAC in PA Patients

**Figure 3:**
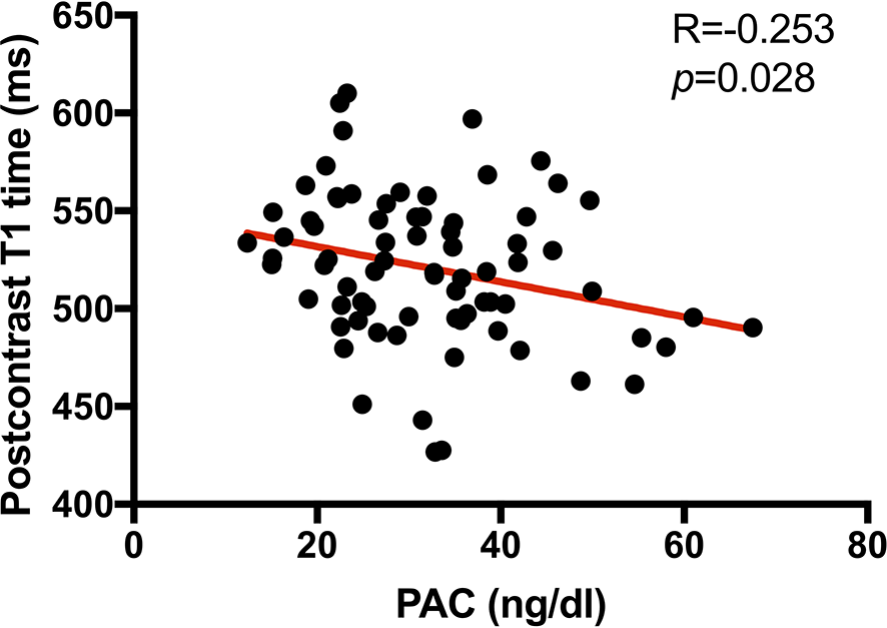
Scatterplot showing the correlation between myocardial postcontrast T1 time (ms) and plasm aldosterone concentration (PAC, ng/dl) in PA patients. Thus, higher PAC was associated with a higher burden of interstitial myocardial collagen deposition (represented by a shorter postcontrast T1 time) in PA patients.

As showed above, the global circumferential PDSR was lower in PA patients than EH patients but not statistically related to PAC. However, Pearson’s analysis (**Figure 3**) showed that PAC was negatively related to postcontrast myocardial T1 time (R = −0.253, p = 0.028).

### Postcontrast Myocardial T1 Time Negatively Related to PAC in PA Patients

Inter-observer variabilities of tissue tracking between two experienced radiologists were minimum, which were shown in **Table 5**. Correlation coefficient r were 0.749–0.957, p <0.001 for all. Intra-observer variabilities for tissue tracking were r = 0.816–0.955, p <0.001 for all. Myocardial tissue tracking was reproducible and reliable.

**Table 5:** Inter-and intra-observer variability of tissue tracking.

	Inter-observer	95%CI	Intra-observer	95%CI
	r (n = 20)		r (n = 20)	
**PS (%)**				
** Radial**	0.909	0.757–0.977	0.955	0.7896–0.980
** Circumferential**	0.874	0.781–0.946	0.823	0.617–0.933
** Longitudinal**	0.802	0.561–0.965	0.932	0.832–0.978
**PSSR (1/s)**				
** Radial**	0.941	0.873–0.983	0.940	0.843–0.982
** Circumferential**	0.939	0.784–0.995	0.887	0.756–0.954
** Longitudinal**	0.750	0.544–0.935	0.849	0.645–0.989
**PDSR (1/s)**				
** Radial**	0.889	0.779–0.961	0.937	0.858–0.977
** Circumferential**	0.957	0.835–0.990	0.816	0.639–0.918
** Longitudinal**	0.749	0.501–0.945	0.878	0.595–0.996

Abbreviations as in **Table 2**.

## Discussion

To the best of our knowledge, this is the first study to demonstrate the relationship between CMR-verified cardiac diastolic dysfunction and diffuse myocardial fibrosis assessed by postcontrast T1 time in PA patients. Although there was no significant difference was found in systolic cardiac functions between PA and EH patients, the global circumferential PDSR was lower, the native-T1 and ECV were higher, and the post-T1 time was shorter in PA patients than in blood pressure matched EH patients. In PA patients, postcontrast T1 time independently related to global circumferential PDSR after adjusting for confounding factors in the multivariate regression analysis. Additionally, the postcontrast T1 time is reversely related to PAC. Our study implies that diffuse myocardial fibrosis, which may be caused by elevated PAC level, affects left ventricular diastolic function in PA patients.

In the present study, we also found that the global circumferential PDSR is a more sensitive indicator for identifying the left ventricular dysfunction in PA patients than ejection fraction, PS, and PSSR. Catena found that PA patients had lower left ventricular diastolic function than EH patients but no systolic function differences with echocardiographic measurements (10), which was similar to our finding. However, Catena (10) and Muiesan (28) also reported greater left ventricular mass in PA patients than in EH patients, but we did not see a significant difference in left ventricular mass in this study. This may be due to our PA patients’ short disease course, which was not long enough to cause a marked increase in left ventricular mass. The median of PA patients’ hypertension duration was 3 years in this study. The PA patients’ hypertension duration was 9.2 ± 8 years in Freel’s study which also showed no significant difference in LV massi between PA and EH patients (17). In comparison, PA patients’ mean hypertension duration was 15 years in Grytaas’ study which reported higher LV massi in PA patients than healthy subjects (15). And the mechanisms of aldosterone-induced LV hypertrophy and aldosterone-induced LV fibrosis were different. The aldosterone promotes LV hypertrophy by activating mineralocorticoid receptors (MRs), extracellular signal-regulated kinase (ERK), c-Jun N-terminal kinase (JNK), and protein kinase C-α (PKC-α) (29). Basic and clinical studies found that aldosterone-induced cardiac fibrosis is associated with the activation of MRs and glucocorticoid receptors (GRs) through genomic and non-genomic pathways (30). Previous echocardiography studies showed a decreased global longitudinal strain in PA patients compared with EH (31, 32). It seems diverse to our finding. However, they found the lower longitudinal strain in PA patients, which showed not significantly different in our study, which may be related to our participant characteristics with a short duration of hypertension. On the other hand, the strain rate would be affected by the heart rate. Our study showed global circumferential PDSR was lower in the PA patients. Still, the difference of PDSR between PA and EH patients was not described in Chen’s and Boulestreau’s study, and additional research is needed to determine the change of PDSR in PA patients. Furthermore, we found a strong correlation (β = 0.963, p <0.001) between the global longitudinal PDSR and averaged across all five layers and the inter-observer and intra-observer variabilities of global longitudinal PDSR were 0.749 and 0.878. The value of global longitudinal PDSR in our study was reliable.

Diastolic dysfunction plays a causative role in the process of cardiac failure (33). In one study that included 6,067 heart failure patients over 15 years, 47% of patients had a preserved ejection fraction, and the morbidity/mortality of preserved ejection fraction heart failure was comparable to that of reduced ejection fraction heart failure (34). In our study, the median duration of hypertension was only three years, but the global circumferential PDSR was decreased in PA patients, indicating that even patients with a short PA course have already developed subclinical cardiomyopathy. Therefore, it is essential to assess cardiac function, especially diastolic function, but not only to ejection fraction in PA patients with a course of more than three years to identify subclinical cardiomyopathy.

Several studies have demonstrated that PA patients have increased fibrosis than healthy volunteers (16, 35) and EH patients (17, 18, 35). However, Gretaas et al. (15) reported a reverse result in his research. In the present study, we found that diffuse myocardial fibrosis represented by postcontrast T1 time was increased in PA patients compared to blood pressure controlled EH patients. Abundant elementary experimental studies supported that aldosterone could promote myocardial fibrosis directly and indirectly. Aldosterone directly increases rat cardiac myofibroblast proliferation by activating Ki-RasA, the MAPK1/2 cascade (36), and insulin-like growth factor-I receptor (37). Aldosterone also has an indirectly profibrogenic function by upregulating inflammation in cardiomyocytes (38) and inhibiting antifibrotic factors, including BNP and ANP (39). Our study showed that the PAC was negatively related to postcontrast myocardial T1 time, which indicates that the diffuse myocardial fibrosis may be caused by high PAC level.

We speculate that the different salt consumption of included patients may explain the different myocardial fibrosis results in PA patients in different research. From a rat model of hyperaldosteronism, the myocardial fibrosis only developed in rats with high salt intake (40), and Ang II type 1 receptor might have some implications in this model (41). The habitual dietary sodium intake of the participants was not recorded in Gretaas’ study (15). However, in Freel’s (17) and our study, patients were recruited in the local population with consuming a salt-rich diet, and myocardial fibrosis was increased. Besides, the serum sodium of PA patients was higher than EH patients in our study.

CMR-verified diffuse myocardial fibrosis was proved to associate with diastolic dysfunction in heart failure (12), hypertrophic cardiomyopathy (13), and diabetic patients (14), but it remains unknown in PA. Su (16) reported increased myocardial fibrosis and left ventricular mass in PA patients compared with healthy controls, but they did not perform a correlation analysis between these two parameters. Our study proved an independent relationship between the global circumferential PDSR and CMR-verified diffuse myocardial fibrosis, suggesting that hyperaldosteronism in PA may contribute to cardiac dysfunction by promoting myocardial fibrosis.

Our study had some limitations. The myocardial biopsy is the gold standard for the diagnosis of cardiomyopathy. Considering its invasiveness and financial cost, we did not have histological evidence of myocardial fibrosis to validate the results of T1 mapping. Furthermore, because this was a single-center study, there is a need for multicenter, large-scale trials to confirm our findings.

## Conclusions

The global circumferential PDSR derived by CMR is decreased, and the diffuse myocardial fibrosis is increased in PA patients compared to those in blood pressure matched EH patients. The severity of cardiac diastolic dysfunction independently relates to the degree of diffuse myocardial fibrosis in PA patients, and the diffuse myocardial fibrosis may be caused by high PAC level.

## Data Availability Statement

The raw data supporting the conclusions of this article will be made available by the authors, without undue reservation.

## Ethics Statement

The studies involving human participants were reviewed and approved by the Ethics Committee of West China Hospital. The patients/participants provided their written informed consent to participate in this study.

## Author Contributions

FZ designed research, acquisition, and analysis of data, writing the manuscript. TW, WW, and SW collection of data. WC analysis of the data. HT, TC, and JS designed research. YR designed the study, revising the manuscript, and final approval of the manuscript submitted. All authors contributed to the article and approved the submitted version.

## Funding

This study was supported by West China Hospital, Sichuan University (Grant Nos. ZYGD18022 and 2018HXFH009), Department of Science and Technology of Sichuan Province (Grant Nos. 2019YJ0040, 2020YFS0123 and 2021YFS0198) and Health Department of Sichuan Province (Grant No. 20PJ047).

## Conflict of Interest

The authors declare that the research was conducted in the absence of any commercial or financial relationships that could be construed as a potential conflict of interest.
